# Sar1, a Novel Regulator of ER-Mitochondrial Contact Sites

**DOI:** 10.1371/journal.pone.0154280

**Published:** 2016-04-21

**Authors:** Karin B. Ackema, Cristina Prescianotto-Baschong, Jürgen Hench, Shyi Chyi Wang, Zhi Hui Chia, Heidi Mergentaler, Fredéric Bard, Stephan Frank, Anne Spang

**Affiliations:** 1 Growth and Development, Biozentrum, University of Basel, 4056 Basel, Switzerland; 2 Division of Neuropathology, Institute of Pathology, University Hospital Basel, 4031 Basel, Switzerland; 3 Institute for Molecular and Cell Biology, Singapore 138673, Singapore; Institute of Molecular and Cell Biology, Biopolis, SINGAPORE

## Abstract

Endoplasmic reticulum (ER)—mitochondrial contact sites play a pivotal role in exchange of lipids and ions between the two organelles. How size and function of these contact sites are regulated remains elusive. Here we report a previously unanticipated, but conserved role of the small GTPase Sar1 in the regulation of ER-mitochondrial contact site size. Activated Sar1 introduces membrane curvature through its N-terminal amphiphatic helix at the ER-mitochondria interphase and thereby reducing contact size. Conversely, the *S*. *cerevisiae* N3-Sar1 mutant, in which curvature induction is decreased, caused an increase in ER-mitochondrial contacts. As a consequence, ER tubules are no longer able to mark the prospective scission site on mitochondria, thereby impairing mitochondrial dynamics. Consistently, blocking mitochondrial fusion partially rescued, whereas deletion of the dynamin-like protein enhanced the phenotype in the *sar1D32G* mutant. We conclude that Sar1 regulates the size of ER-mitochondria contact sites through its effects on membrane curvature.

## Introduction

Lipids are transported and exchanged between cellular compartments through either vesicular transport or inter-organellar contact sites. In the first case, a piece of membrane is pinched off from a donor compartment and fuses with a target compartment. Through fusion, the lipids in the transport vesicles mix with those of the target membrane. In the case of inter-organellar contact sites, ions and lipids are directly transferred from one compartment to the next. Thus communication between two organelles occurs through vesicle- and non-vesicle mediated transport mechanisms.

Vesicular transport from the endoplasmic reticulum (ER) to the Golgi apparatus is mediated by the COPII coat. In a first step, the small GTPase Sar1 is activated by its GEF Sec12 at the ER and then the Sec23/24 complex is recruited. This complex is stabilized by interaction with cargo molecules to be exported from the ER. Finally, the Sec13/31 complex binds and promotes membrane deformation resulting in a bud that is still attached to the membrane. Scission of vesicles is supported by the N-terminal amphiphatic helix of Sar1, which is essential for the Sar1 liposome tubulation activity *in vitro* [1–4].

The ER also exchanges material with other organelles through non-vesicular pathways, most notably the mitochondria. Phosphatidylserine (PS) is transported from the ER to mitochondria and converted there into phosphatidylethanolamine (PE). PE is then transported back to the ER from where it populates other compartments. In metazoans, in addition to lipid transport, Ca^2+^ exchange occurs at ER-mitochondria contacts. How contact sites are formed and how they are regulated is still largely unexplored. At least in yeast, a tethering complex, ERMES, helps to connect mitochondria and ER [5]. Yet, ERMES may not be directly involved in the exchange of material but rather ensures the close proximity of both organelles [6]. The ERMES components do not seem to be conserved in metazoans, but tethering structures are likely to exist, even though their protein composition might be different.

A third, presumably independent, role for ER contacting mitochondria is that ER tubules mark future scission sites on mitochondria [7]. Studies in yeast provide evidence that this process may ensure correct mtDNA distribution [8].

Reticulons have been shown to positively regulate lipid exchange between mitochondria and ER [9]. We were wondering whether another protein with membrane shaping activity, Sar1, could also influence ER-mitochondria contacts. Here we report that Sar1 negatively regulates the size of ER-mitochondrial contact sites by providing ER membrane curvature and/or bilayer asymmetry.

## Results and Discussion

### Sar1 is essential for mitochondrial function and morphology in yeast

**Fig 1:**
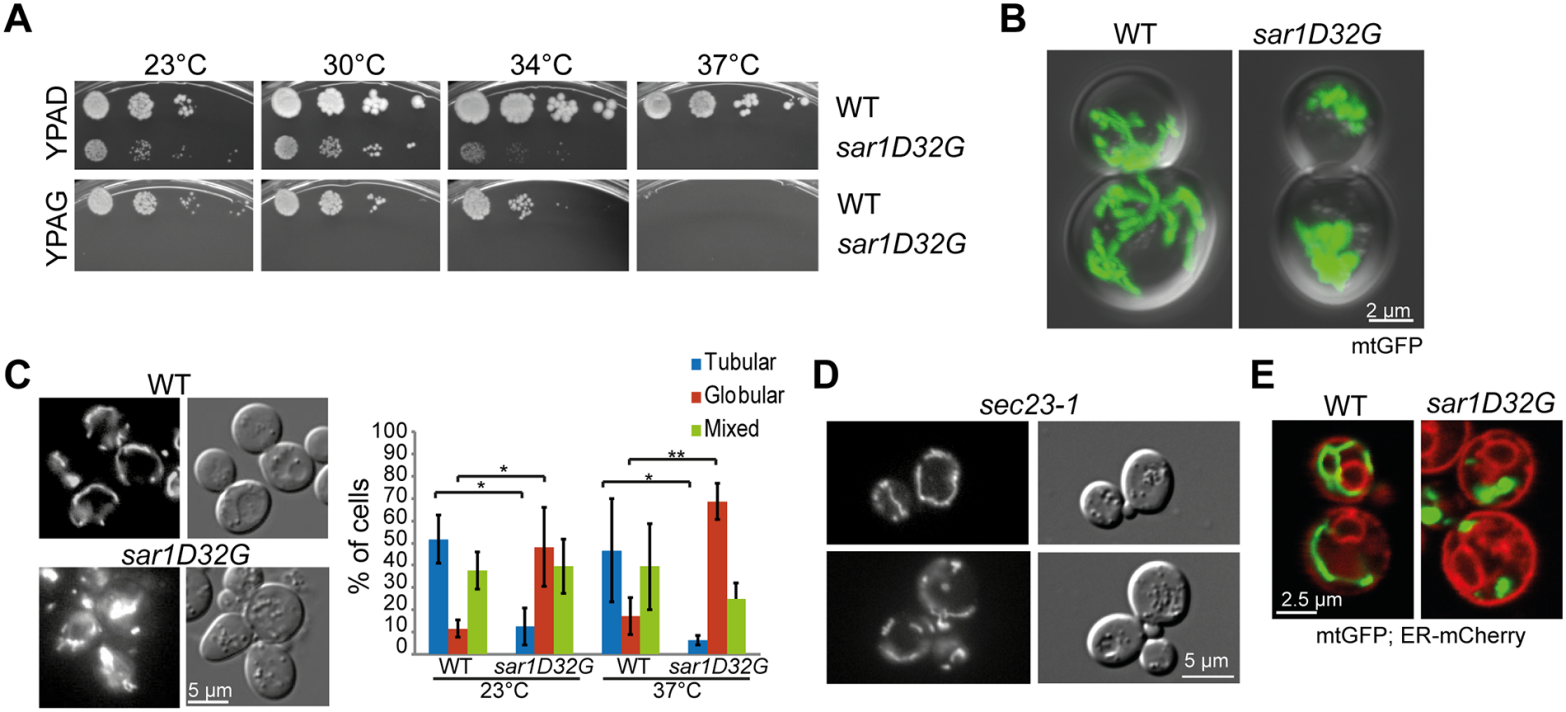
Sar1 is required for mitochondrial function and morphology. (**A**) Growth assay of wild-type and *sar1D32G* cells on YP plates complemented with adenine and glucose or adenine and glycerol. *sar1D32G* is unable to grow on glycerol plates, even at the permissive temperatures. (**B**) The mitochondrial network is perturbed in *sar1D32G* cells. Live confocal microscopy of wild-type and *sar1D32G* cells expressing mt-GFP, shifted to 37°C for 1 h. Maximal projections of DIC and GFP channels. (**C**) *sar1D32G* mutant cells have more globular mitochondria, and less tubular networks. Epifluorescence images of wild-type and mutant cells expressing mt-GFP; and quantification of the phenotypes. The data are derived from at least 3 independent experiments in which > 100 cells were analyzed per strain and temperature. Error bars: standard deviation; p-values: * < 0.05; ** < 0.005. (**D**) The mitochondrial network is maintained in a Sar1GAP Sec23 mutant. *sec23-1* cells expressing mt-GFP were shifted to 37°C (restrictive temperature) for 1 h before imaging. (**E**) The ER morphology is altered in *sar1D32G* cells. Cells expressing chromosomally tagged Pho88-mCherry and mt-GFP were shifted to 37°C for 1 h. *sar1D32G* cells show a more extended ER, but mitochondria are still located in close proximity to ER membranes.

Reticulons are involved in efficient lipid exchange between ER and mitochondria [9]. We hypothesized that other proteins with membrane shaping activity may also play a role in the function and/or assembly of ER-mitochondrial contact sites. Next to the reticulons, the small GTPase Sar1 has been shown to have membrane-shaping activity [1–3]. If Sar1 was involved in the regulation of ER mitochondrial contact sites, one would expect that in a *sar1* mutant mitochondrial function should be impaired. To test this hypothesis, we performed a growth test with the temperature-sensitive GDP-restricted *sar1D32G* mutant [10, 11] on plates containing glycerol as sole carbon source. Yeast requires functional mitochondria to be able to grow on non-fermentable carbon sources such as glycerol. The *sar1D32G* mutant was unable to grow on glycerol plates even at permissive temperatures (23°C and 30°C) (Fig 1A), indicating a defect in mitochondrial function. To corroborate these findings, we assessed mitochondrial morphology in the *sar1D32G* mutant. While wild-type cells displayed an elaborate mitochondrial network, clusters of mitochondrial fragments were observed in the *sar1* mutant (Fig 1B and 1C). Consistent with the lack of growth on glycerol plates at the permissive temperature, globular mitochondrial structures were already observed at 23°C (Fig 1C). These clusters were not present in a mutant of the essential COPII component *SEC23* after shift to the restrictive temperature (*sec23-1*, Fig 1D), suggesting a secretion-independent role of Sar1 in mitochondrial function. Moreover, the general ER morphology is also not driving the mitochondrial morphology defects in *sar1D32G* because blocking ER exit by any mutant in COPII causes dilated ER [12], yet *sec23-1* cells maintained mitochondrial networks under the same conditions. Furthermore, although a mutant in the small GTPase Arf1, another essential component of the early secretory pathway, *arf1-11*, displays dilated ER and aberrant mitochondria, the mitochondrial phenotype is very different from the one observed in *sar1D32G* and caused by the failure of Arf1-11 to recruit Cdc48 to mitochondria [13]. While *CDC48* overexpression alleviated the *arf1-11* mitochondrial phenotype [13], we did not observed any rescue in *sar1D32G* (data not shown). Our data indicate that Sar1 is required for mitochondrial function in a more direct way than just through secondary effects caused by blocking ER-Golgi transport.

### *sar1D32G* increases the size of ER-mitochondrial contacts

**Fig 2:**
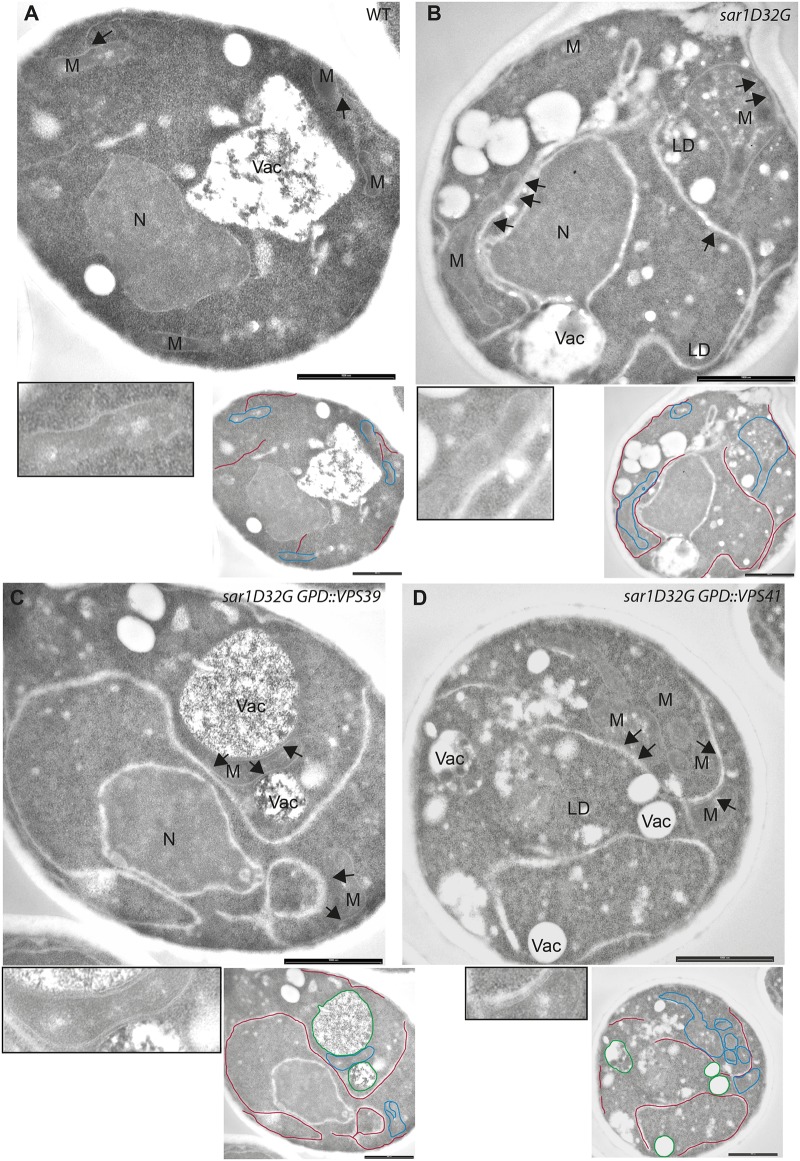
Loss of Sar1 function increases ER-mitochondria contact sites. Transmission electron microscopy images of cells of WT (**A**), *sar1D32G* (**B**), *sar1D32G* overexpressing *VPS39* (**C**) *sar1D32G* overexpressing *VPS41* (**D**) after incubation at 1 h at 37°C. The bars represent 1 μm. N: nucleus; M: mitochondria; Vac: vacuole; LD: lipid droplet. The arrows point to regions of ER mitochondria contacts. The insets represent a two-fold magnification of a region of interest. Relevant ER membranes, mitochondria and vacuoles were traced in red, blue and green, respectively, in the smaller images.

To gain deeper insights into the mitochondrial phenotype induced by *sar1D32G*, we performed an ultrastructural analysis using electron microscopy. Unexpectedly, we observed a strong increase in ER-mitochondrial contacts in *sar1D32G* cells (compare Fig 2A and 2B). The length of the contacts detected in micrographs extended well beyond 1 μm. Consistent with impaired mitochondrial function in *sar1D32G* mutant cells, cristae were aberrant or even absent and replaced by amorphous material and lipid accumulations. Thus, in *sar1D32G* mutant cells, the size of the contact sites might no longer be controlled. Alternatively, the increase in contact site size in the *sar1* mutant could potentially be due to a compensation for non-functional contact sites. To demonstrate that the effect of *sar1D32G* on ER-mitochondrial contact sites was specific, we attempted to rescue the phenotype by overexpressing wild type Sar1. Indeed, contact site length was reduced and ER expansion largely rescued (S1 Fig). However, mitochondria remained affected, as they now appeared to be thinner with inner membrane defects. This effect correlated with Sar1 overexpression because increased Sar1 levels in the wild-type background equally changed mitochondrial appearance. This phenotype was reminiscent of the one observed in MICOS mutants [14]. Moreover, we analyzed *sec23-1* cells after shift to the restrictive temperature by electron microscopy. Even though the ER was expanded, mitochondria were not affected in the same way as in *sar1D32G* mutant cells (data not shown). Thus, inactivating as well as overexpressing Sar1 impairs mitochondrial morphology, indicating that there is indeed a direct link between Sar1 function and mitochondrial morphology.

### Overexpression of vCLAMP does not alleviate the *sar1D32G* mutant phenotype

We tested next, whether the increase of contact site area in *sar1D32G* cells is due to a compensation mechanism for aberrant lipid exchange. Lipid exchange between ER and mitochondria is not limited to the direct route, but may also occur via vacuolar contacts with both organelles. Recently the v-CLAMP tethering complex was proposed to allow lipid transport between mitochondria and vacuole and may be particularly important when ER-mitochondrial contact sites are perturbed [15, 16]. Therefore, we tested whether overexpression of the v-CLAMP component Vps39 could rescue the *sar1D32G* phenotype. Overexpression of *VPS39* caused an increase in tethering of the mitochondria to vacuoles, without rescuing mitochondrial lipid accumulations to any appreciable extent (Fig 2C). Likewise, the mitochondrial morphology defect, as judged by live cell imaging, was not rescued (S2 Fig). Consistent with previous reports, the mitochondrial tethering function of Vps39 was independent of its role in the HOPS complex because overexpression of the HOPS-specific *VPS41* [17] did not have any appreciable effect on mitochondrial localization (Fig 2D). Thus, as overexpression of the v-CLAMP component *VPS39* is unable to rescue the *sar1D32G* phenotype, defects in lipid transport may not be the primary cause of defective mitochondria in *sar1D32G*. These data suggest that the lipid exchange per se might still be functional at ER-mitochondrial contact sites, and that the aberrant structures and lipid accumulations might be due to other impairments of the ER-mitochondrial interaction. In agreement with this notion, deletion of either the phosphatidylserine decarboxylase (*PSD1*) or *CRD1*, a gene essential for cardiolipin synthesis strongly exacerbated the *sar1D32G* phenotype (data not shown). Therefore we speculate that the control of ER-mitochondrial contact size might be severely affected in the *sar1* mutant.

### Disruption of the N-terminal amphipathic helix in Sar1 increases ER-mitochondria contacts

**Fig 3:**
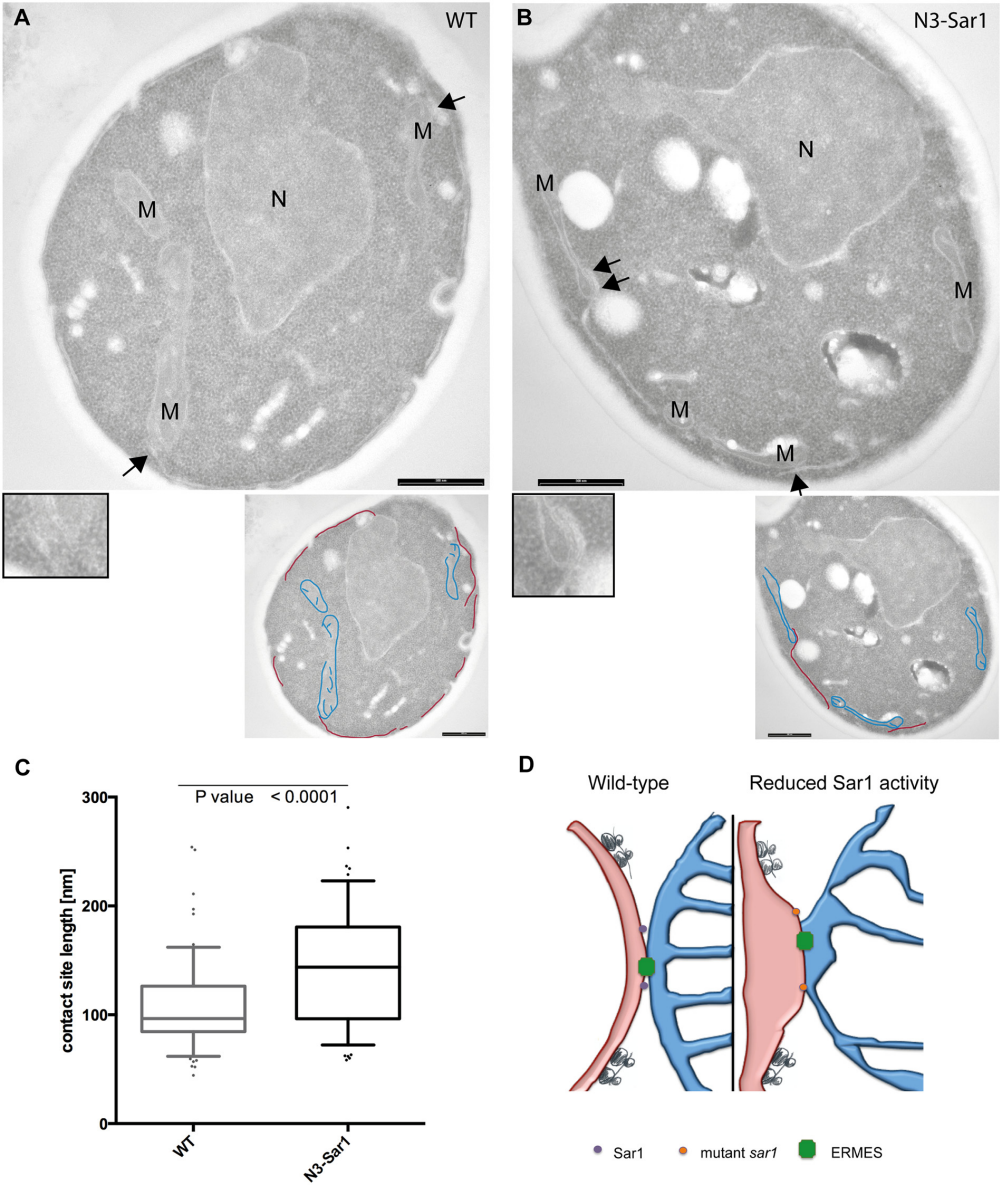
The N3-Sar1 mutant increases ER-mitochondria contacts site length. Transmission electron microscopy images of (**A**) wild type and (**B**) N3-sar1. The bars in (A) and (B) correspond to 1 μm. N: nucleus; M: mitochondria. Arrows point to regions of ER-mitochondria contacts. The insets display a 2x magnification of a region of interest. Relevant ER membranes and mitochondria were traced in red and blue, respectively, in the smaller images. (**C**) Quantification of the phenotype presented in (A) and (B). > 60 contacts were measured/genotype from two independent embeddings. An unpaired, two tailed T-test was used for statistical analysis. The whiskers represent 10–90% of the data. The line in the box indicates the mean. (**D**) Model on Sar1 function at ER-mitochondrial contact sites. For explanation see text.

So far we have shown that the *sar1D32G* mutant maintains extensive ER-mitochondria contacts, consistent with a model in which the Sar1 membrane-shaping activity would control ER-mitochondrial contact site area. To test this hypothesis, we took advantage of another *sar1* mutant, N3-Sar1. Sar1 has a N-terminal amphipathic helix, which promotes COPII vesicle scission *in vivo* and is required for the liposome tubulation activity of Sar1 *in vitro* [1–3]. This activity can be reduced through specific mutations in the N-terminal helix. The N3-Sar1 mutant protein is less potent in tubulating liposomes than the wild-type protein [1]. However, *in vivo* no growth phenotype was detected, only a kinetic delay in the maturation of Gas1 [1]. We determined the apparent size of ER-mitochondrial contacts in thin sections analyzed by electron microscopy in wild type and N3-Sar1 mutant cells (compare Fig 3A and 3B). The length of ER-mitochondria contacts was considerably increased in the N3-Sar1 mutant cells (Fig 3C), indicating that the contact size area must be extended in the mutant compared to wild type. In the N3-Sar1 mutant only two residues of the 23 amino acids constituting the amphipathic helix are mutated [1]; a Sar1 mutant in which the entire helix is deleted (Δ23Sar1) is unable to bind to membranes and is therefore not activated by the ER-resident Sar1GEF Sec12. Thus, by only very moderately affecting Sar1 function, not even perturbing GTPase activity, we observe a significant increase in contact site size. Our data suggest that Sar1 regulates ER-mitochondrial contact size through local changes in membrane curvature. We propose that Sar1 membrane contact through the amphipathic helix induces curvature and/or generates local membrane asymmetry. Lipids and ions are exchanged through ER-mitochondrial contacts suggesting that the opposing membranes are in close contact. To achieve functional contacts the local membrane curvature might be relatively low. Thus, Sar1 activation at the edge of ER-mitochondria contact sites will increase the membrane curvature and thereby locally reduce contacts between ER and mitochondria (Fig 3D). Alternatively to the Sar1-induced membrane curvature changes, the membrane rigidity might be influenced at contact sites. The Sar1 amphipathic helix might change the membrane environment, thus reducing the rigidity to allow COPII vesicles to pinch off or—in our case—to reduce ER-mitochondrial contacts [18]. Both possibilities are not mutually exclusive and very difficult to distinguish *in vivo*. Differences in membrane rigidity would presumably be accompanied by local changes in lipid composition and potentially also changes in protein localization. Sar1 might be ideally suited to regulate contacts depending on the cellular demands. Other ER-shaping molecules like the reticulons are always present in the membrane and through their v-shaped conformation, curvature regulation would have to be regulated by changes in protein concentration (transcription/translation and degradation). In contrast, Sar1 is activated by the ER-resident protein Sec12, which is present along the entire the ER [19, 20]. Thus, local activation of Sar1 at ER-mitochondrial contacts would provide the means by which their size could rapidly be modulated.

### Sar1 is present in ER-mitochondria enriched membrane fractions

In our model we predict that Sar1 would be present at the edges of ER-mitochondrial contact sites, or even being part of them. To test this assumption, we prepared highly enriched mitochondria fractions [13, 21, 22] and probed them for the presence of Sar1 (S3 Fig). Because of ER-mitochondrial tethering, mitochondrial fractions contain always also ER. Still, Sar1 was present in the mitochondrial fraction, and the signal for Sar1 was even stronger than in the bulk of the ER fractions (S3B Fig, compare Fr. 8 to Fr. 4 and 5). Since this fraction also contained ER and no guanine nucleotide exchange factor for Sar1 (Sar1GEF) has been identified on mitochondria to date, we assume that Sar1 is present on ER membranes that are tethered to mitochondria. We conclude that Sar1 is present at ER-mitochondrial contacts.

### Sar1 is not essential for ER-mitochondria tethering

The results presented so far indicate that Sar1 is present at ER-mitochondrial contact sites to negatively regulate their size. A prediction from this model is that ER-mitochondrial tethering would be independent of Sar1 function and hence not perturbed in the Sar1 mutant. In yeast, the ERMES complex acts as ER-mitochondrial tether [5]. Therefore, we next checked whether ERMES complex localization was altered in *sar1D32G* cells. Expression of the mitochondrially-localized GFP-Mdm34 did not result in a difference in localization between wild-type and mutant cells (S3C Fig). Likewise, the localization of the ER part of the tether, Mmm1, was unaffected by the *sar1D32G* mutation (S3D Fig). Consistent with other reports [5, 23, 24], we detected 2 to 5 Mmm1-positive structures per cell. Given that deletion of any single ERMES component is essential for complex assembly [5], we assume that ERMES is assembled correctly in *sar1D32G* mutant cells.

Next, we attempted to rescue the *sar1D32G* phenotype by expression of an artificial ER-mitochondrial tether. ChiMERA consists of two transmembrane domains (TMDs) joined by GFP [5]. One of the TMDs spans the ER and the other the outer mitochondrial membrane, thereby promoting close proximity of the two organelles. This construct was used successfully in a screen in which rescue of mitochondrial morphology was assessed [5]. However, expression of ChiMERA did not rescue the *sar1D32G*-induced phenotype (S3E Fig), further supporting that the ERMES tethering complex is correctly assembled and functional in *sar1D32G* mutant cells. Given the extensive lipid exchange between both organelles -also in light of the extensive contacts recently reported between ER and plasma membrane [25, 26]- we propose that ERMES may serve as a relatively stable contact, from which less stable ER-mitochondria contacts could expand. Interestingly, deletion of various reticulons negatively affects ER-mitochondrial contacts [9]. This phenotype, however, is rescued by ChiMERA, indicating that the reticulons help to maintain tethering by the ERMES complex. The size of less stable contacts would be dependent on the metabolic status including energy and lipid demands of the cell. This scenario seems plausible also considering the recent discovery of the ER membrane protein complex (EMC) as an additional tether to ERMES [27] and of Lam6, general component of mitochondrial contact sites [28]. Thus, two types of membrane-shaping proteins, the reticulons and Sar1, might be involved the regulation of ER-mitochondrial contacts.

### Sar1 is involved in the regulation of mitochondrial dynamics

**Fig 4:**
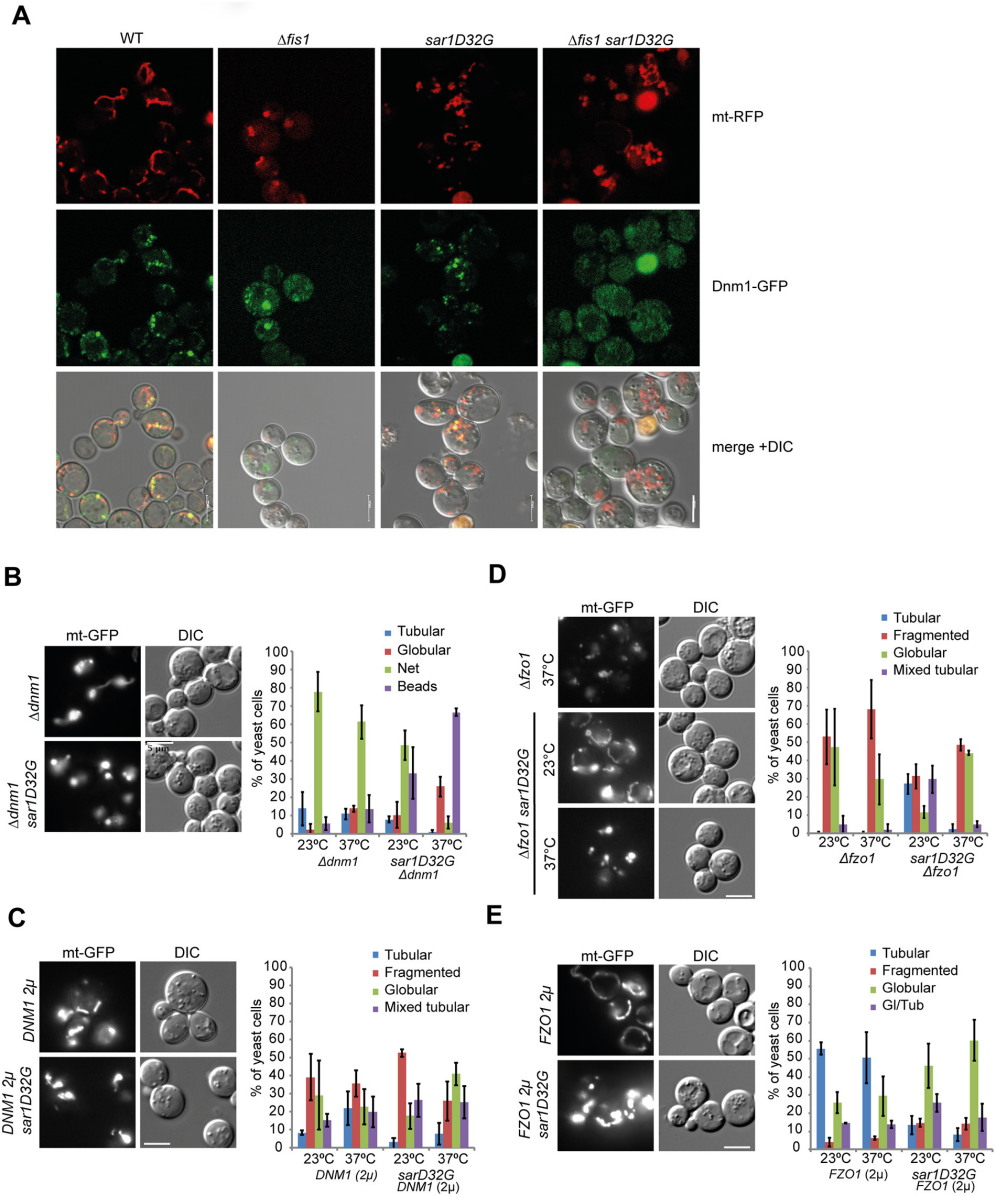
Sar1 plays a role in mitochondrial dynamics. (**A**) Dnm1 recruitment to mitochondria is independent of Sar1 function. Confocal sections of different strains expressing mt-RFP and Dmn1-GFP after shift to 37°C for 1 hr. The merge is shown in together with the DIC channel. (**B-E**) Live imaging of strains expressing mt-GFP at 23°C and 37°C. Epistasis experiments of *sar1D32G* and the isogenic wild-type together with (**B**) *Δdnm1*, (**D**) *Δfzo1*, overexpression of (**C**) *DNM1*, or (**E**) *FZO1*. (**B**) *Δdnm1* exacerbates the *sar1D32G* phenotype. (**C**) *DNM1* overexpression did not affect the *sar1D32G* phenotype and vice versa. (**D**) *sar1D32G* partially rescues the *Δfzo1* phenotype at 23°C. (**E**) *sar1D32G* counteracts the tubular network formation in *FZO1* overexpressing cells. For each of three independent experiments, we scored about 100 cells/genotype. Standard deviation is given.

If the Sar1 membrane-shaping activity is important for ER-mitochondrial contacts, a second type of encounter might also depend on Sar1. ER tubules contact mitochondria and mark the future mitochondrial fission site [7]. We asked whether *sar1D32G* would affect mitochondrial dynamics. First, we determined whether the mitochondrial fission mediator, the dynamin-like GTPase Dnm1, was recruited to mitochondria in *sar1D32G*. Dnm1 localized to mitochondria in both wild-type and *sar1D32G* mutant cells, while in *Δfis1* cells Dnm1 was no longer recruited to mitochondria (Fig 4A), consistent with Fis1 being essential for Dnm1 recruitment to mitochondria [29]. However, when we combined *Δdnm1* with *sar1D32G*, we observed an exacerbation of the individual phenotypes (Fig 4B), similar to what we observed in *Δfis1 sar1D32G* cells (Fig 4A). Overexpression of *DNM1* did not affect the *sar1D32G* clusters, and the *sar1D32G* did not alleviate the *DNM1* overexpression-induced fragmentation (Fig 4C). Our data suggest that Sar1D32G either negatively influences fission or positively affects fusion. Thus, a reduction in marking of future scission sites by an ER tubule may further affect mitochondrial fission dynamics.

Dnm1 function is counteracted by the mitofusin Fzo1 [30]. If Sar1D32G has a negative effect on fission, *sar1D32G* should alleviate the fragmented mitochondria phenotype of *Δfzo1* mutants. As reported previously, *Δfzo1* cells contain fragmented mitochondria [31, 32]. Combining *Δfzo1* with *sar1D32G* reduced the severity of the *Δfzo1* fragmentation phenotype at 23°C (Fig 4D). In addition, *sar1D32G* counteracted the mitochondrial network formation promoted by overexpression of Fzo1 (Fig 4E). Taken together, our data are consistent with *sar1D32G* negatively affecting mitochondrial fission. These results are consistent with a model in which Sar1 function plays a role in the regulation of ER-mitochondrial contact size and mitochondrial fission and fusion dynamics. The partial rescue of the mitochondrial fragmentation in *Δfzo1* by *sar1D32G* might be due to increased ER-mitochondrial contact surface. We speculate that this increased contact surface could have an inhibitory effect on Dnm1-mediated scission.

### Loss of Sar1 function causes mitochondrial phenotypes in metazoans

**Fig 5:**
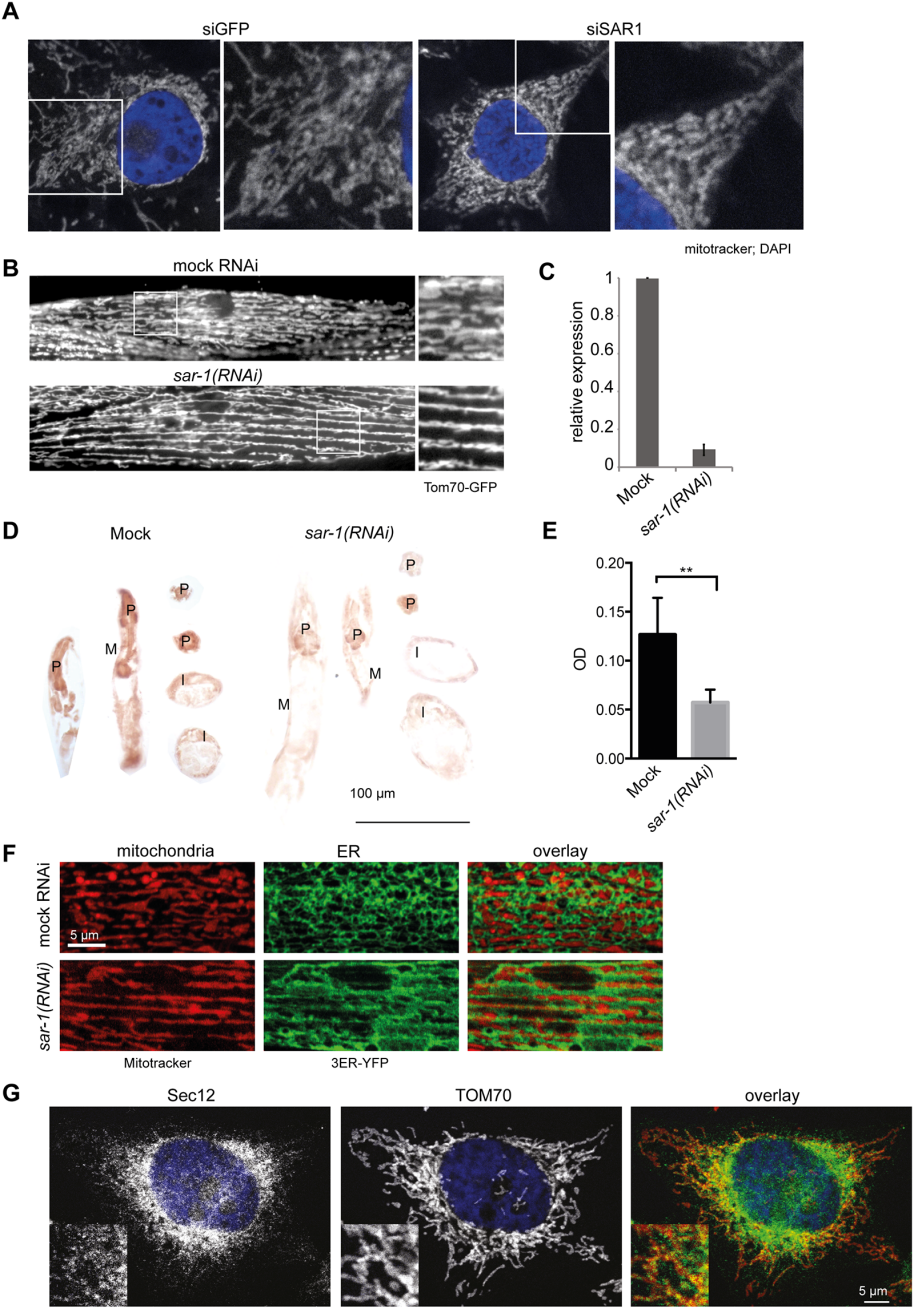
The effect of loss of Sar1 function on mitochondria is conserved in metazoans. (**A**) Mitochondrial morphology is affected by siRNA-based knockdown of SAR1 in HeLa cells. Confocal microscopy of fixed cells. Mitochondria were visualized with Mitotracker, and the nucleus was stained with DAPI. (**B**) Live-cell imaging of Tom70-GFP in *C*. *elegans* muscle cells. In mock treated worms, the mitochondria form typical tubular structures along the contractile apparatus, which are connected with each other forming a network. Those connections are absent in *sar-1(RNAi)* muscle cells, and the mitochondria are collapsed into straight lines. (**C**) *sar-1(RNAi)* is efficient. qPCR analysis of mock treated and *sar-1(RNAi)* worms. (**D**) The enzymatic activity of cytochrome c oxidase is reduced in *sar-1(RNAi)* compared to mock treated animals. P: pharynx, M: muscle, I: intestine. (**E**) Quantification of the resulting optical density (OD) in the pharynx. Mock: n = 13, *sar-1(RNAi)*: n = 12. Error bars represent standard deviation. p < 0.005 (t-test). (**F**) The ER is expanded in *sar-1(RNAi)* muscle cells. Maximal projections of mitotracker and the ER marker. (**G**) Sec12 is at ER mitochondrial contact sites. Immunofluorescence analysis of HeLa cells with antibodies against Sec12 and TOM70. The signal between Sec12 and TOM70 is partially overlapping. A representative image is shown from 3 independent experiments in which at least 30 cells were analyzed. The inlet represents a two-fold magnification of an area of interest.

To assess whether the role of Sar1 in mitochondrial morphology and function is conserved throughout metazoans, we knocked down its mammalian homolog SARA1 in HeLa cells. The mitochondrial network was aberrant in the knockdown when compared to mock treated cells (Fig 5A). Likewise, mitochondrial morphology was severely perturbed in *C*. *elegans* body wall muscle cells (Fig 5B, S4 Fig), in which mitochondria are normally arranged between neighboring actin bundles but also go across actin bundles to form a loosely connected network [13, 33]. In *sar-1(RNAi)* cells, mitochondria appeared to be elongated resulting in a highly regular striped pattern along actin bundles with reduced cross-over and a diminished network (Fig 5B, S4 Fig). The efficiency of the knockdown was determined by qPCR (Fig 5C). Strikingly the *sar-1(RNAi)* phenotype was very different from the hyperconnected mitochondrial network observed in *arf-1*.*2(RNAi)* body wall muscle cells [13]. Consistent with the mitochondrial phenotype, *sar-1(RNAi)* strongly reduced mitochondrial function as determined by staining for cytochrome c oxidase activity (Fig 5D and 5E) [34]. Like in the yeast *sar1D32G* mutant, ER morphology was severely altered in *sar-1(RNAi)* body wall muscle cells (Figs 1E and 5F). This is a common phenomenon in early secretory pathway mutants in yeast [12]. However, ER defects are not necessarily linked to a mitochondrial phenotype, as in the *sec23-1* mutant, the mitochondrial network was still maintained (Fig 1D). Moreover, knockdown of *arf-1*.*2* affected ER morphology in oocytes but not in body wall muscle cells [13]. Thus, there is no strict correlation between ER and mitochondrial morphology and phenotypes. We next assessed the localization of mitochondria with respect to the ER in *sar-1(RNAi)* muscle cells. Similar to mock treated cells, the ER surrounded mitochondria, with both organelles maintaining contact (Fig 5F).

Our model suggests that Sar1 activation could occur close to ER-mitochondria contact sites. In *Saccharomyces cerevisiae*, the Sar1GEF Sec12 is distributed over the entire ER [20], making it likely that Sar1 would be recruited to contact sites. To provide additional evidence, we determined the localization of Sec12 with respect to mitochondria in mammalian cells. There, Sec12 is localized to discrete places within the ER network, presumably ER exit sites [35].

Consistent with Sec12 function at ER exit sites, the majority of the Sec12 signal did not overlap with mitochondria. However, a subpopulation of Sec12 co-localized with mitochondrial TOM20 (Fig 5G), suggesting that Sar1 was activated by Sec12 at ER-mitochondrial contact sites.

Taken together our data suggest that Sar1 plays a conserved role in mitochondrial morphology and function. We propose that Sar1 acts as a negative regulator of ER-mitochondrial contact size, in a conserved process, presumably independent of the well-established function in COPII vesicle generation at the ER.

## Materials and Methods

### Yeast Methods and Plasmids

Standard genetic techniques were employed [36]. Transformations and deletions were performed as described [37, 38]. For drop assays, cells were grown to log phase YPD. Cells were adjusted to OD_600_ 0.1, followed by 3 serial 10-fold dilutions, spotted onto YP plates with either 2% glucose or 3% glycerol + 1 g/l adenine and incubated at various temperatures. The mtGFP plasmids pVT100U-mtGFP (URA3) and PYX142-mtGFP (LEU2) were provided by B. Westermann [39]. pYX142-mtRFPm (LEU2) was made in the lab of K. Okamoto and provided by J. Shaw [40]. pRS416GPD-mtRFP (URA3) was made by inserting the mtRFP fragment derived from pYX142-mtRFP into pRS416GPD using EcoRI and XhoI. pRS426-FZO1 (B1155) and pRS426-DNM1 (B1472) overexpression plasmids were provided by J. Shaw. Yeast strains are specified in S1 Table. For microscopy, cells were grown O/N at 23°C in selective medium supplemented with adenine (1 g/l). Cultures were diluted to early log phase and grown at 23°C for 4 h in YPAD. Half of each culture was shifted to 37°C for 1 h before analysis by microscopy. For quantification, approximately 100 cells were counted in each individual experiment, which was repeated at least twice. For ultrastructural analysis, cells were fixed and treated as described previously [41]. Mitochondria were purified from yeast cultures grown in YP glycerol according to [42].

### *C*. *elegans* strains and plasmids

*C*. *elegans* was cultured and maintained as described previously [43] at 20°C. The pmyo-3::Tom70::GFP plasmid [33] was co-injected together with pRF4 (*rol-6(su1006)*) [44].

### RNAi and siRNA experiments

RNAi was performed as described [45]. NGM plates containing 1.5 mM IPTG and 25 μg/ml carbenicillin were inoculated with RNAi bacteria and induced for ~12 h at room temperature. Eggs or larvae were cultured at 20°C or 25°C until adulthood. For muscle cells, all RNAi strains were fed for 2 days at 20°C starting from L2/L3 larvae. The enzymatic COX assays and the mitotracker labeling were performed as described before [13, 34]. Enzymatic COX histochemistry is an *in situ* assay that quantifies potential activity of the COX protein through 3,3'-diaminobenzidine staining intensity. If performed with an on-slide control tissue—in our case mock control *C*. *elegans* worms—signal intensity is determined relatively to this reference. Absolute quantification by enzymatic histochemistry is not feasible, as reactions are individually terminated once the reference tissue reaches visible staining levels [34].

siRNA assays were performed as described before [13]. Three days after siRNA transfection, cells were stained with 0.5 μM of MitoTracker for 30 min at 37°C and followed by fixation in PBS containing 4% paraformaldehyde, 4% sucrose and permeabilization with 0.05% NP-40/PBS. DNA was visualized with DAPI.

### Imaging

For live imaging, adult hermaphrodites were mounted in M9 without anesthetics between the slide and coverslip using Vaseline^®^ at the edges to function as a spacer. Fixed samples were mounted on uncoated slides in anti-fade reagent CitiFluor (Citifluor Ltd., UK) with a small rim of Vaseline^®^ on the side.

Yeast expressing Dnm1-GFP/mtRFP, and yeast expressing Pho88-mCherry/mtGFP were imaged on an Andor Revolution spinning disk confocal system (Andor Technologies, Northern Ireland) mounted onto an IX-81 inverted microscope (Olympus, Center Valley, PA), equipped with iXon^EM^+ EMCCD camera (Andor Technologies). Mitotracker/Tom70-GFP and yeast mtGFP experiments were imaged on a Leica SP5-II-Matrix confocal microscope with a HCX PLAN APO lambda blue 63x oil objective (Leica Microsystems, Germany). The worms were imaged using the high resonance scanner at 8000 Hz, 2.5x Zoom and 16x line average. Yeast mtGFP was imaged with normal scanner settings at 1000 Hz, 15 zoom and 8x line average. LAS AF version 2.6.0.7266 software was used to control hardware and to acquire images. Epifluorescence images for quantification of yeast and worm phenotypes, and muscle actin staining were taken on a Zeiss Axioplan 2 microscope equipped with a Zeiss Axio Cam MRm camera (Carl Zeiss, Germany) and a Plan Apochromat 63x/NA1.40 oil objective. Zeiss Axiovision 3.1 software was used to control hardware and to acquire images. Images of HeLa cells were taken with a Zeiss LSM700 confocal microscope with 10x 63 magnification and 2x zoom. ImageJ was used for post-processing and analysis of all images.

### qPCR

pPCRs were performed as described previously [13]. The primers for sar-1 were sar-1 Q1 fw 5’-GTCCCACTTCATTTCCCCCT-3’, sar-1 Q1 re 5’-CGAACTGCAACATTCCGAAGC-3’. Expression levels were normalized to cdc-42 [46]. Fold differences were calculated using the delta-delta Ct method, corrected for PCR efficiency [47]. The average knockdown was determined based on three independent experiments.

### Sec12 immunofluorescence

Hela cells were seeded onto glass coverslips in 24-well dishes (Nunc, Denmark). After overnight incubation, cells were fixed with 4% paraformaldehyde, 4% sucrose in D-PBS for 10 min, washed with D-PBS and permeabilized with 0.2% Triton X-100 for a further 10 min. The cells were then stained with Sec12 (Novus Biologicals, LLC (#NBP1-87056)) and TOM20 primary antibody (Santa Cruz Biotechnology (#sc-17764)) diluted in 2% FBS in D-PBS overnight. Cells were subsequently washed three times for 5 min with 2% FBS in D-PBS and stained for 20 min with secondary anti-rabbit conjugated with 488nm fluorophore, anti-mouse conjugated with 594nm fluorophore and Hoechst 33342 (all Thermo Fisher Scientific) diluted in 2% FBS in D-PBS. Cells were mounted onto glass slides using FluorSave (Merck) and imaged at room temperature using an inverted confocal microscope (Zeiss LSM700) with a 100x objective under Immersol oil (Zeiss). Images were acquired at 100x magnification and processed using Zeiss Zen software.

## Supporting Information

S1 Fig Overexpression of *SAR1* causes mitochondrial phenotypes. Ultrastructural analysis of cells shifted for 1 h to the restrictive temperature overexpressing *SAR1* from a multicopy plasmid in comparison to untransformed cells. While the *sar1D32G* phenotype was partially rescued by the overexpression of wild-type *SAR1*, mitochondria became thinner and inner mitochondrial membranes were aberrant in both wild-type and mutant cells. The inlets are twofold magnifications of an area of interest in the micrographs. The bars represent 1 μm.
(TIF)

S2 Fig Overexpression of *VPS39* does not alleviate the *sar1D32G* mitochondrial phenotype. Live-cell imaging of cells of diverse strains expressing mt-GFP.
(TIF)

S3 Fig Characterization of the ER-mitochondria contact sites. (**A**) Sar1 fractionates with heavy membranes. Immunoblot of samples from a differential centrifugation. (**B**) Sar1 co-fractionates with mitochondria and ER-mitochondria contacts. The P12,000 fraction from (A) was loaded on a sucrose gradient. Fractions of the gradient after centrifugation were subjected to immunoblot analysis. Pgk1 is a cytoplasmic protein, Sec61 is ER-localized, and Por1 is the mitochondrial porin. (**C-E**) The ERMES complex is functional in *sar1D32G* mutants. (**C**) Mdm34 localizes into spots in *sar1D32G* mutants. Life cell imaging of Mdm34-GFP and Pho88-mCherry (ER-mCherry) in WT and *sar1D32G* cells after incubation for 1 h at 37°C. (**D**) Mmm1-RFP localizes into spots in *sar1D32G* mutant cells. Live-cell imaging of WT and *sar1D32G* cells expressing Mmm1-RFP after incubation for 1 h at 37°C. The bars in (A) and (B) correspond to 5 μm. (**E**) chiMERA does not rescue the *sar1D32G* phenotype. The morphology of mitochondria was assessed by mt-RFP with or without expression of chimera (GFP channel) after incubation to 37°C for 1 h.
(TIF)

S4 Fig *sar-1(RNAi)* leads to a tubular mitochondrial pattern. Three more examples of the *sar-1(RNAi)* phenotype displayed in Fig 5.
(TIF)

S1 Table Yeast strains used in this study. (DOCX)
