# Supplementary figures and images for: Sar1, a Novel Regulator of ER-Mitochondrial Contact Sites
Source: PLoS One. 2016 Apr 21;11(4):e0154280. doi: 10.1371/journal.pone.0154280 (PMC4839682; doi:10.1371/journal.pone.0154280)

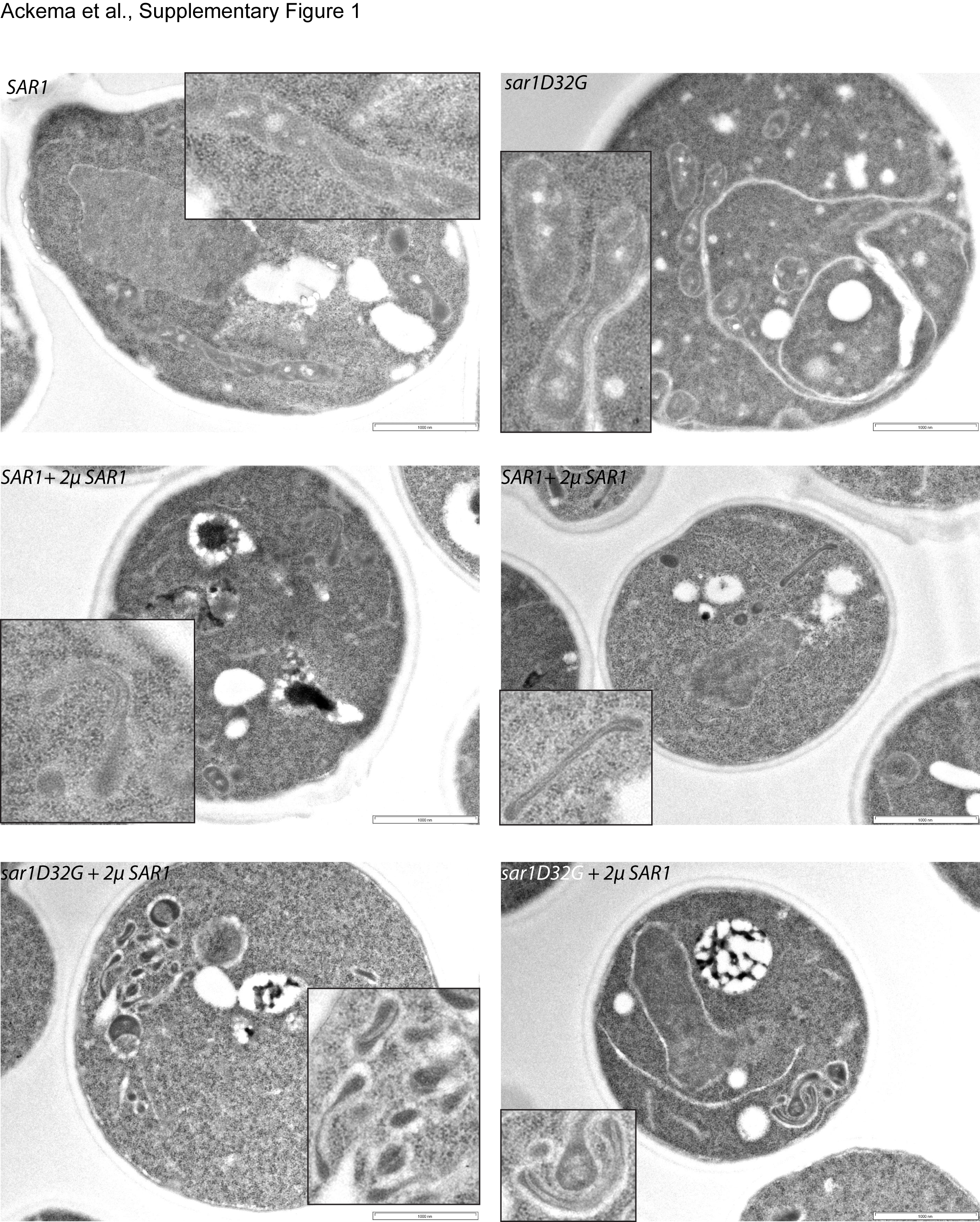

Supplement: S1 Fig — Ultrastructural analysis of cells shifted for 1 h to the restrictive temperature overexpressing SAR1 from a multicopy plasmid in comparison to untransformed cells. While the sar1D32G phenotype was partially rescued by the overexpression of wild-type SAR1, mitochondria became thinner and inner mitochondrial membranes were aberrant in both wild-type and mutant cells. The inlets are twofold magnifications of an area of interest in the micrographs. The bars represent 1 μm. (TIF) [file pone.0154280.s001.tif]

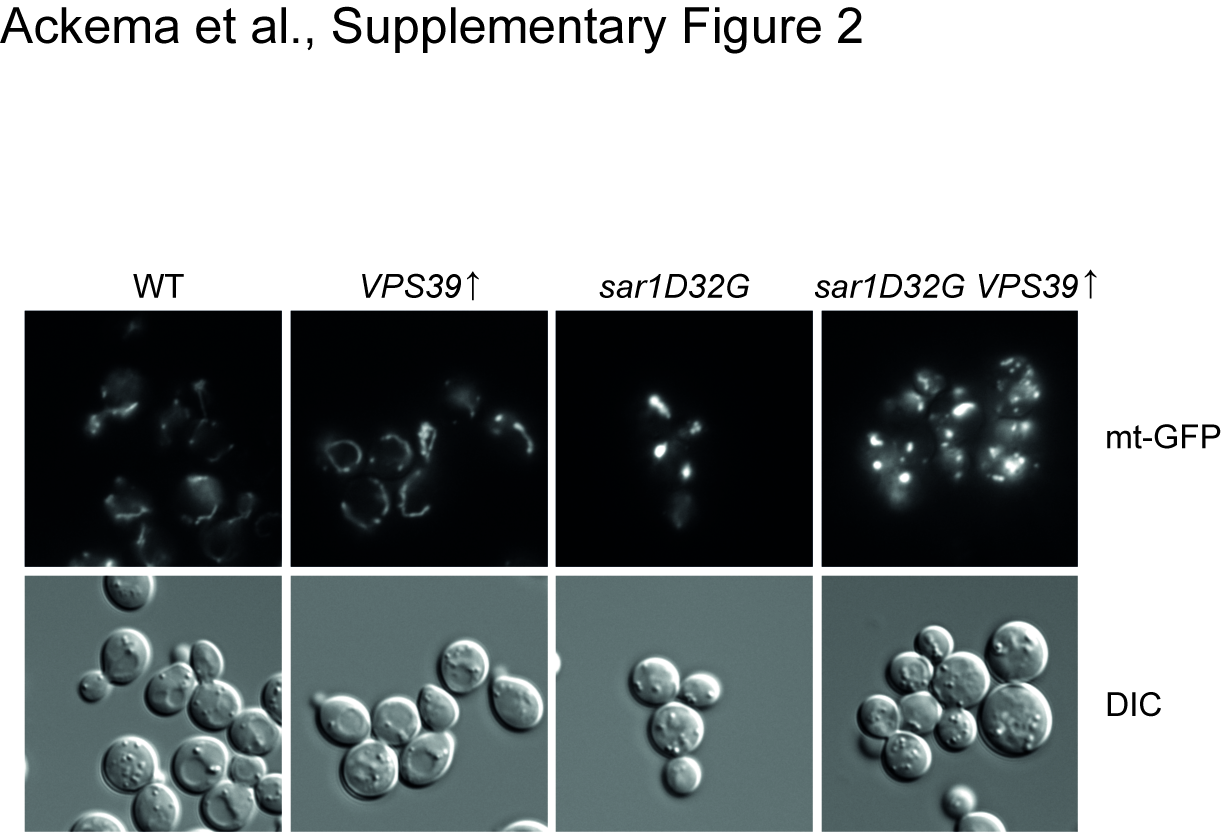

Supplement: S2 Fig — Live-cell imaging of cells of diverse strains expressing mt-GFP. (TIF) [file pone.0154280.s002.tif]

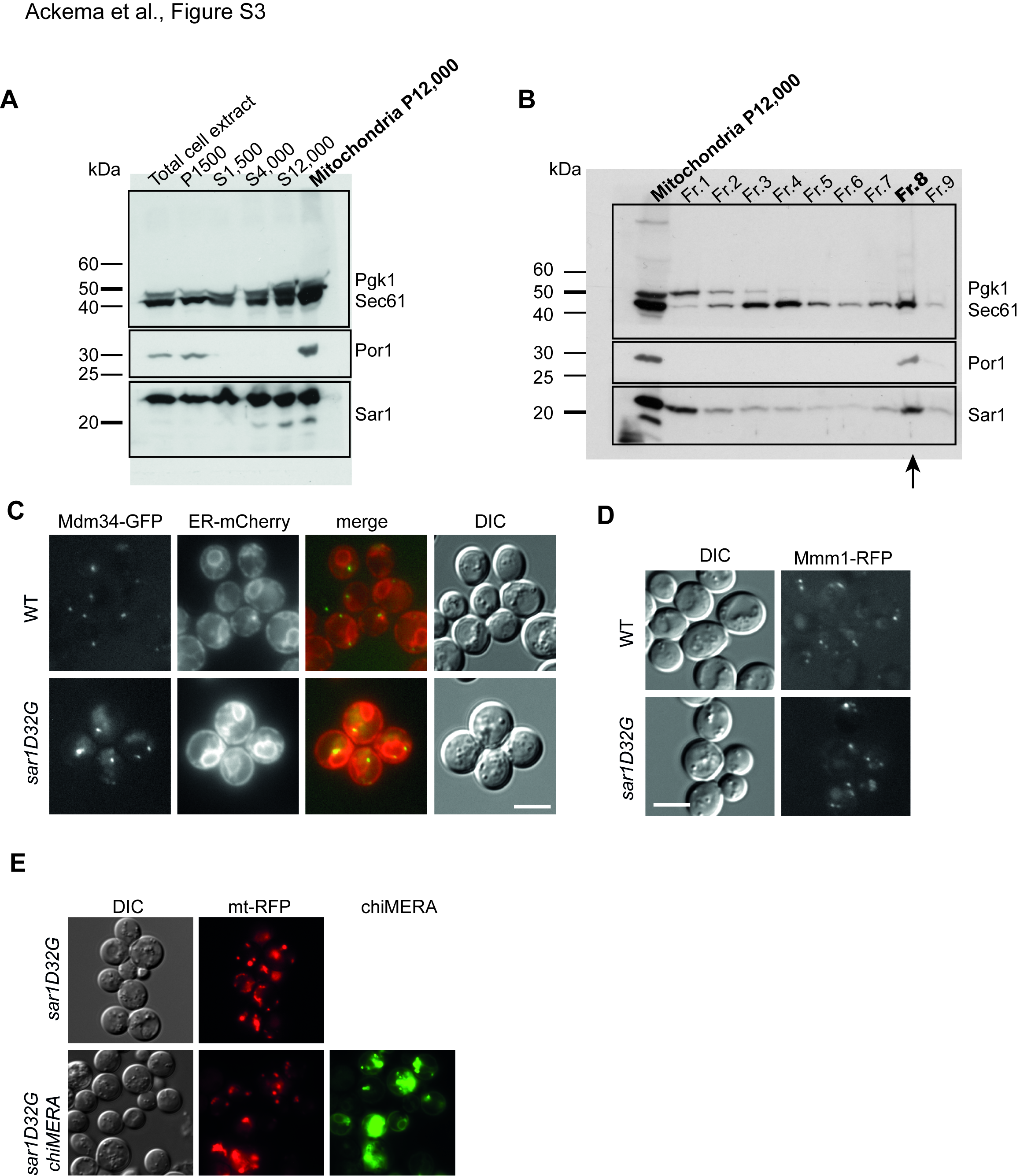

Supplement: S3 Fig — (A) Sar1 fractionates with heavy membranes. Immunoblot of samples from a differential centrifugation. (B) Sar1 co-fractionates with mitochondria and ER-mitochondria contacts. The P12,000 fraction from (A) was loaded on a sucrose gradient. Fractions of the gradient after centrifugation were subjected to immunoblot analysis. Pgk1 is a cytoplasmic protein, Sec61 is ER-localized, and Por1 is the mitochondrial porin. (C-E) The ERMES complex is functional in sar1D32G mutants. (C) Mdm34 localizes into spots in sar1D32G mutants. Life cell imaging of Mdm34-GFP and Pho88-mCherry (ER-mCherry) in WT and sar1D32G cells after incubation for 1 h at 37°C. (D) Mmm1-RFP localizes into spots in sar1D32G mutant cells. Live-cell imaging of WT and sar1D32G cells expressing Mmm1-RFP after incubation for 1 h at 37°C. The bars in (A) and (B) correspond to 5 μm. (E) chiMERA does not rescue the sar1D32G phenotype. The morphology of mitochondria was assessed by mt-RFP with or without expression of chimera (GFP channel) after incubation to 37°C for 1 h. (TIF) [file pone.0154280.s003.tif]

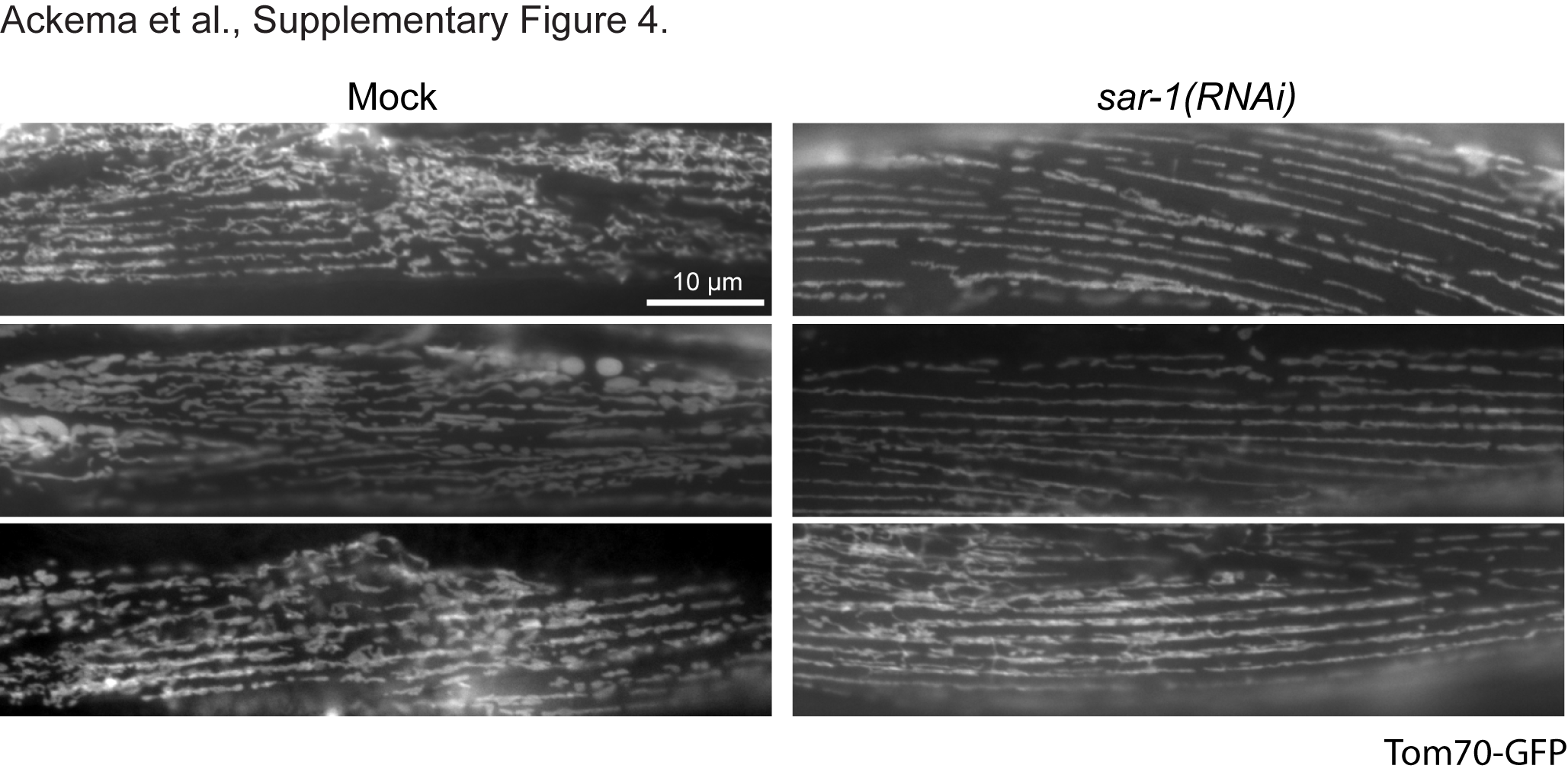

Supplement: S4 Fig — Three more examples of the sar-1(RNAi) phenotype displayed in Fig 5. (TIF) [file pone.0154280.s004.tif]
