# Supplementary material for: Sar1, a Novel Regulator of ER-Mitochondrial Contact Sites
Source: PLoS One. 2016 Apr 21;11(4):e0154280. doi: 10.1371/journal.pone.0154280 (PMC4839682; doi:10.1371/journal.pone.0154280)
Supplement: S1 Table — (DOCX) [file pone.0154280.s005.docx]

**SUPPLEMENTARY TABLE S1**

| **Table S1**. Yeast strains used in this paper | | |
| --- | --- | --- |
| Strain | Genotype | Source |
| YPH499 | MATa *ura3-52 leu2-*D*1 trp1-*D*63 his3-*D*200 lys2-801 ade2-101* | Sikorski and Hieter (1989) |
| YPH500 | MATα *ura3-52 leu2-*D*1 trp1-*D*63 his3-*D*200 lys2-801 ade2-101* | Sikorski and Hieter (1989) |
| Sar1-WT | MATα *ura3 lys2 trp1 leu2 his2 ade2 SAR1::HIS3PEP4::ADE2* | K. Sato |
| Sar1-D32G | MATα *ura3 lys2 trp1 leu2 his2 ade2 SAR1::HIS3PEP4::ADE2*  pMYY3-1 (YcP [sar1-D32G TRP1]) | K. Sato |
| YAS4038 | MATα *ura3 lys2 trp1 leu2 his2 ade2 SAR1::HIS3PEP4::ADE2 PHO88::PHO88-3x mCherry-Kan* | This Study |
| YAS4039 | MATα *ura3 lys2 trp1 leu2 his2 ade2 SAR1::HIS3PEP4::ADE2*  pMYY3-1 (YcP [sar1-D32G TRP1]) *PHO88::PHO88-3x mCherry-Kan* | This Study |
| YAS4040 | MATa *ura3 lys2 trp1 leu2 arf1 ::HIS3 arf2::HIS3 ade2::ARF1::ADE2 fis1::Kan* | This study |
| LMY0073 | MATa *ura3-52 leu2-*D*1 trp1-*D*63 his3-*D*200 lys2-801 ade2-101 sar1::.KanMX pRS305Sar1WT-2* | Lee et al., 2005 |
| LMY0076 | MATa *ura3-52 leu2-*D*1 trp1-*D*63 his3-*D*200 lys2-801 ade2-101 sar1::.KanMX pRS305Sar1-22-5* | Lee et al., 2005 |
